# Quantitative Analysis for Lung Disease on Thin-Section CT

**DOI:** 10.3390/diagnostics13182988

**Published:** 2023-09-18

**Authors:** Tae Iwasawa, Shoichiro Matsushita, Mariko Hirayama, Tomohisa Baba, Takashi Ogura

**Affiliations:** 1 Department of Radiology, Kanagawa Cardiovascular & Respiratory Center, 6-16-1 Tomioka-higashi, Kanazawa-ku, Yokohama 236-0051, Japan; m.sho160@gmail.com (S.M.); nrk25998@yahoo.co.jp (M.H.); 2 Department of Respiratory Medicine, Kanagawa Cardiovascular & Respiratory Center, 6-16-1 Tomioka-higashi, Kanazawa-ku, Yokohama 236-0051, Japan; baba@kanagawa-junko.jp (T.B.); takaoguogu@gmail.com (T.O.)

**Keywords:** computed tomography, image reconstruction, artificial intelligence, densitometry, lung diseases, interstitial, pulmonary disease, chronic obstructive

## Abstract

Thin-section computed tomography (CT) is widely employed not only for assessing morphology but also for evaluating respiratory function. Three-dimensional images obtained from thin-section CT provide precise measurements of lung, airway, and vessel volumes. These volumetric indices are correlated with traditional pulmonary function tests (PFT). CT also generates lung histograms. The volume ratio of areas with low and high attenuation correlates with PFT results. These quantitative image analyses have been utilized to investigate the early stages and disease progression of diffuse lung diseases, leading to the development of novel concepts such as pre-chronic obstructive pulmonary disease (pre-COPD) and interstitial lung abnormalities. Quantitative analysis proved particularly valuable during the COVID-19 pandemic when clinical evaluations were limited. In this review, we introduce CT analysis methods and explore their clinical applications in the context of various lung diseases. We also highlight technological advances, including images with matrices of 1024 × 1024 and slice thicknesses of 0.25 mm, which enhance the accuracy of these analyses.

## 1. Introduction

**Figure 1:**
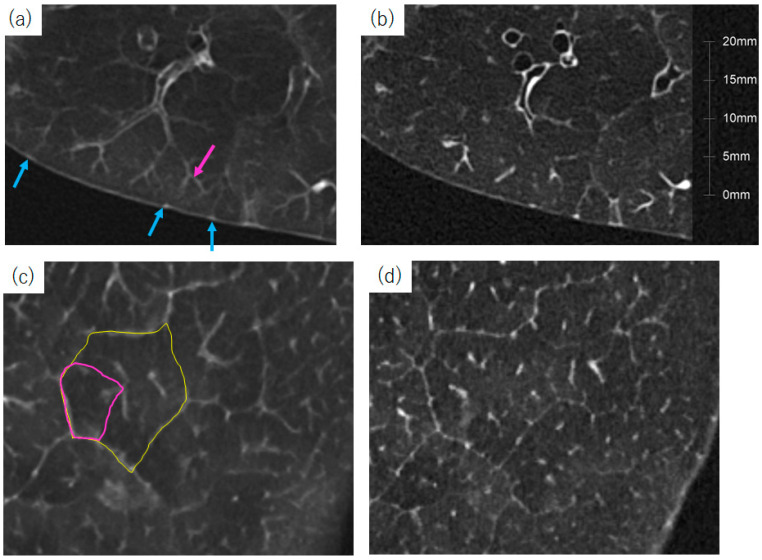
Thin-section CT of a cadaver’s lung with 1024 × 1024 matrices and 0.25 mm of slice thickness. (**a**,**b**) tangential and (**c**,**d**) parallel images to the lung surface. (**a**,**c**) Maximal intensity projection (MIP) images with 2 mm thickness and (**b**,**d**) their original images. These images are 1024 × 1024, with a 0.25 mm slice thickness. Terminal bronchioles are clearly visible on MIP images (a, pink arrows). The small interlobular veins can also be seen (blue arrows). Based on these peripheral structures, Reid’s secondary lobule is recognized (pink polygon). Miller’s secondary lobule can also be observed (yellow polygon).

**Figure 2:**
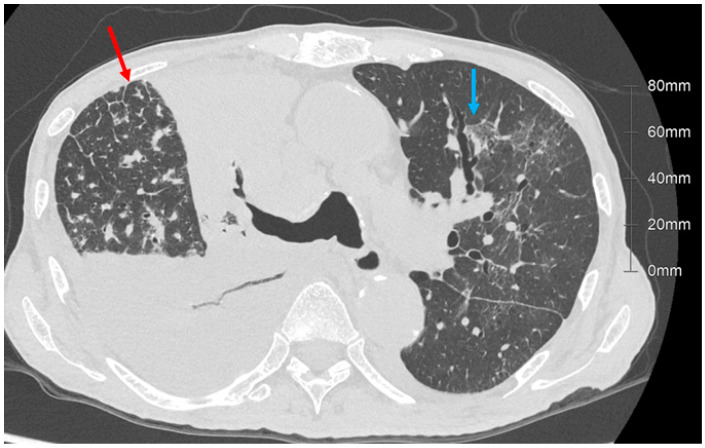
Axial images from a 71-year-old man with lung cancer and COVID-19 pneumonia. In the right lung, there is nodular interlobular septal thickening (red arrow) due to lymphangitis carcinomatosis. In the left lung, there are ground glass opacities (GGO) due to COVID-19 pneumonia (blue arrow). The size of the secondary lobule with GGO is smaller relative to the normal area.

Thin-section computed tomography (CT) is widely used for diagnoses of lung diseases. In particular, the recognition of lung abnormalities relative to the structures of the secondary lobule is fundamental for the diagnoses of diffuse lung diseases [1]. There are two definitions of the secondary pulmonary lobule: Miller’s and Reid’s. Miller’s secondary pulmonary lobules are surrounded by connective tissue septae [1]. Reid’s secondary lobules were defined by Reid based on the branching pattern of peripheral bronchioles [2,3]. The recently developed high-resolution CT technique with 1024 × 1024 matrices and 0.25 mm slice thickness can provide information on peripheral airway [4], and, therefore, Reid’s secondary lobule can be recognized on these high-resolution images (Figure 1 and Figure 2) [5]. The lobules, as defined by Reid, exhibit a nearly uniform size, measuring approximately 1 cm throughout the entirety of the lung. This is particularly important to understand underlying pathophysiological mechanisms. A reduction in the dimensions of secondary lobules implies a decrease in the alveolar volume available for efficient gas transfer. For instance, more compact secondary lobules are commonly observed in severe cases of COVID-19, in which oxygen therapy is administered [5,6]. This particular instance underscores the vital role of information such as volume derived from images for the accurate estimation of lung function. Within this review, we present the most common techniques employed in evaluating respiratory function through the use of thin-section CT scans.

## 2. Overview of Densitometric Analyses

Table 1 summarizes the methods for analyzing respiratory function based on thin-section CT. There are two main types of analysis: those that use attenuation values and those that evaluate the volumes of bronchi, blood vessels, and the lungs.

One of the most widely used quantitative analyses is probably the quantification of emphysema based on attenuation values. Emphysema is histologically defined as a condition of the lungs characterized by abnormal, permanent enlargement of airspaces distal to the terminal bronchiole, accompanied by the destruction of their walls without evident fibrosis [35]. The pixel value on CT is based on the attenuation value of X-rays measured in Hounsfield units (HU; scaled X-ray attenuation coefficient) of a voxel, where air should be −1000 HU, and water should be 0 HU. Thus, emphysema is characterized by the presence of areas of abnormally low attenuation on CT. Using this principle, Müller et al. developed a method known as “density mask” to quantitatively evaluate areas below a certain threshold, empirically defined as emphysema-like [8]. Thresholds of −910 HU and −950 HU have been used to quantify mild and severe emphysema, respectively [8], and this analysis has been histopathologically validated [9,36,37]. This method has been adopted in patient selection for interventional studies [38], gene associations [39,40], and associations with respiratory morbidity [41]. Other indices, including mean lung attenuation (MLA) and attenuation value histograms (Perc15), have also been utilized in patients with chronic obstructive pulmonary disease (COPD) [19] (Table 1). 

Using a similar approach, high-attenuation areas (HAAs) can be utilized as markers of interstitial lung disease (ILD). Increased HAA levels are associated with reduced forced vital capacity (FVC) in patients with collagen vascular diseases [13,14]. Histogram indices, such as skewness, kurtosis, and MLA, have also been correlated with FVC in idiopathic pulmonary fibrosis (IPF) [18,42]. 

## 3. Technical Considerations for Densitometric Analyses

### 3.1. Radiation Dose

Before discussing the usefulness of quantitative image analysis in each disease, some technical aspects should be considered. 

The most important drawback of CT is radiation exposure. Sakane et al. found no detectable effects of low-dose CT (with median effective dose of 1.5 mSv) on human DNA in peripheral blood samples. However, double-stranded DNA breaks and chromosomal aberrations increase after standard-dose examinations [43]. Therefore, dose reduction is preferred, particularly for repeated functional lung imaging. Low-dose CT can provide accurate attenuation values for lung density measurements, similar to standard doses [44]; however, the problem of image noise still remains [45]. There are several solutions to this issue. Photon-counting CT can reduce radiation exposure while, at the same time, maintaining image quality, offering a potential solution [46,47].

### 3.2. Reconstruction Methods

**Figure 3:**
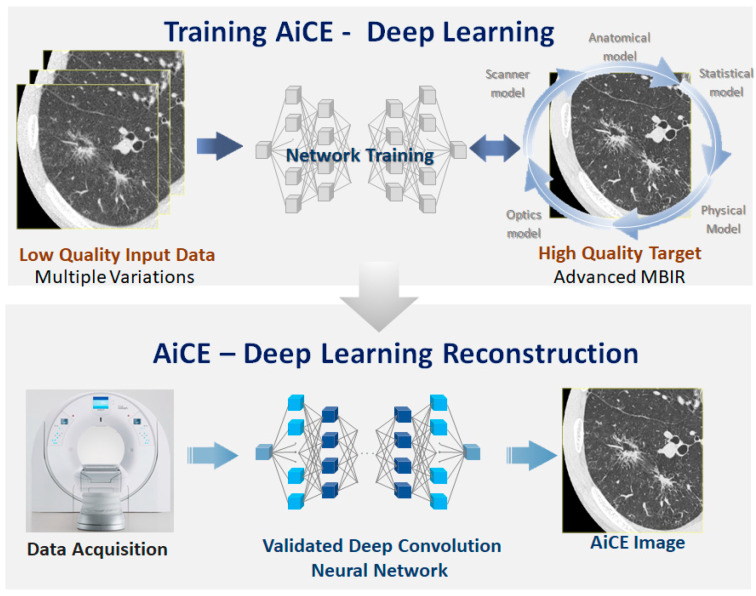
Scheme of a DLR, the Advanced Intelligent Clear-IQ Engine (AiCE, Canon Medical Systems). Top: training process, and bottom: DLR process. A deep convolutional neural network is first trained using high-quality images reconstructed via MBIR as the training target. Once trained, the network is validated and applied to actual data. The algorithm is based on a neural network trained with patient data, where lower-dose hybrid iterative reconstruction images are used as input, and routine-dose full MBIR images serve as the ground truth. Utilizing MBIR as the ground truth not only aids in noise reduction but also helps reduce artifacts by modeling system optics, system physics, scanner statistical properties, and human anatomy. An actual image reconstructed using AiCE is shown in Figure 4. Recently, a super resolution DLR known as PIQE (Canon Medical Systems Corporation) has been commercialized, incorporating deep learning-based super-resolution technology to improve spatial resolution. In PIQE, the network is trained using ultra-high-resolution data of 1024 × 1024 matrix size, paired with downsampled, simulated normalized resolution data. PIQE-reconstructed images are shown in Figure 5. Abbreviations: DLR, deep learning reconstruction; MBIR, model-based iterative reconstruction; and PIQE, Precise IQ Engine.

**Figure 4:**
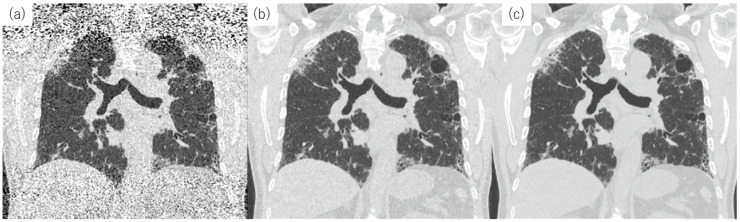
Coronal reconstruction images of low-dose CT obtained from a 63-year-old man with idiopathic pulmonary fibrosis. The estimated radiation exposure was 1.14 mSv. The following reconstruction methods were used: (**a**) FBP. Marked artifacts are visible in the FBP image; (**b**) HIR (AIDR). Artifacts are reduced in the HIR image, but noise remains; and (**c**) DLR (AiCE). Emphysema and interstitial lesions are clearly visible in the DLR image. Abbreviations: AiCE, Advanced Intelligent Clear-IQ Engine; AIDR, adaptive iterative dose reduction; DLR, deep learning reconstruction; FBP, filtered back projection; and HIR, hybrid iterative reconstruction.

**Figure 5:**
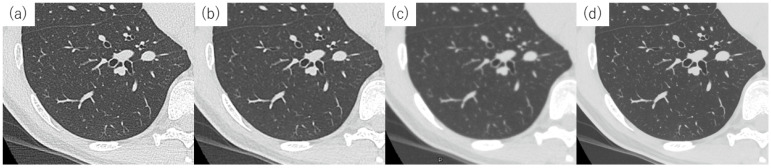
Axial images of standard-dose CT obtained from a 43-year-old woman with normal respiratory function. (**a**) FBP. (**b**) HIR using AIDR with a sharp kernel and (**c**) with a smooth kernel. (**d**) PIQE. The spatial resolution is 512 × 512 for the matrix size and 0.5 mm slice thickness in images (**a**–**c**). In image (**d**), the matrix size is 1024 × 1024, and the slice thickness is 0.5 mm. With PIQE, the sharpness of the pulmonary vessels and bronchi is similar to that of HIR with a sharp kernel, whereas the noise is the lowest among the four images. Abbreviations: AIDR, adaptive iterative dose reduction; CT, computed tomography; DLR, deep learning reconstruction; FBP, filtered back projection; HIR, hybrid iterative reconstruction; and PIQE, Precise IQ Engine.

**Figure 6:**
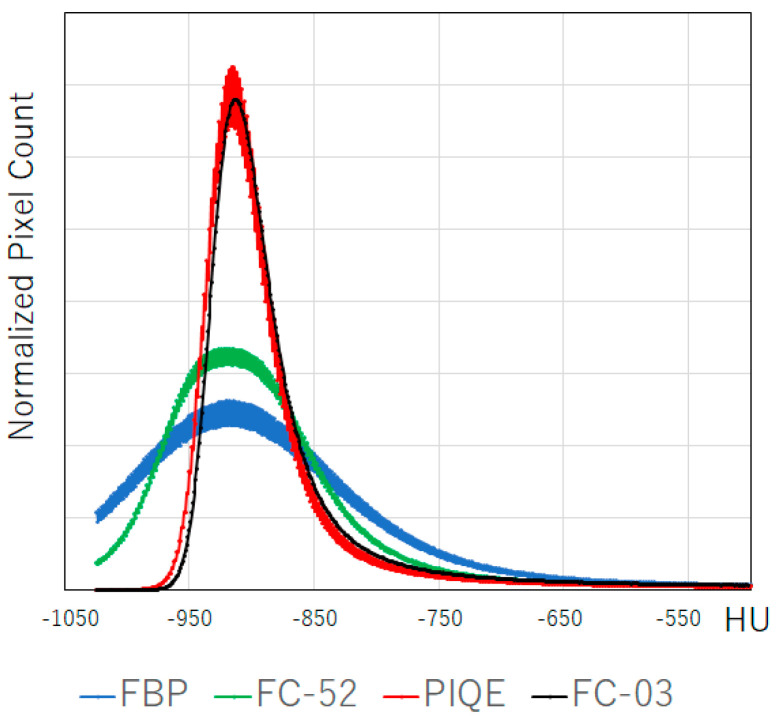
Histogram of CT attenuation values of the lung in different reconstruction images, featuring the patient described in Figure 5. The histogram represents the entire lung and was reconstructed using the following methods: FBP (blue), HIR (AIDR) with a sharp kernel (green), HIR (AIDR) with a smooth kernel (black), and PIQE (red). The histograms of HIR with a sharp kernel and FBP are broad owing to noise, resulting in a large %LAA, as shown in Figure 7. The histogram of PIQE is as sharp as that corresponding to HIR with a smooth kernel, even with an increase in spatial resolution. Abbreviations: AIDR, adaptive iterative dose reduction; FBP, filtered back projection; HIR, hybrid iterative reconstruction; HU, Hounsfield unit; LAA, low-attenuation area; and PIQE, Precise IQ Engine.

**Figure 7:**
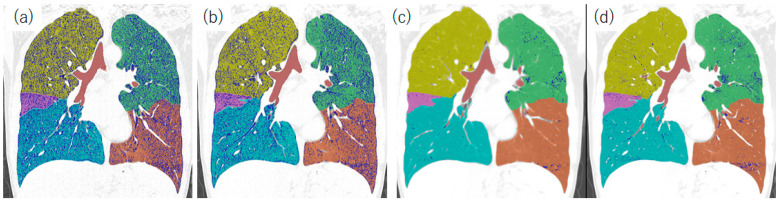
%LAA results in different reconstruction images featuring the patients described in Figure 5. Blue color shows pixels with values under −950 HU. (**a**) FBP, (**b**) HIR (AIDR, sharp kernel), (**c**) HIR (AIDR, smooth kernel), and (**d**) PIQE. A large number of blue pixels can be seen in FBP (**a**) and AIDR with a sharp kernel (**b**) image. The lungs are segmented into lobes with the following color codes: yellow, the right upper lobe; pink, the right middle lobe, cyan, the right lower lobe; green, the left upper lobe; and brown, the left lower lobe. Abbreviations: AIDR, adaptive iterative dose reduction; FBP, filtered back projection; HIR, hybrid iterative reconstruction; HU, Hounsfield unit; LAA, low-attenuation area; and PIQE, Precise IQ Engine.

Improvement in image reconstruction techniques may offer another way to reduce image noise (Figure 3 and Figure 4). Filtered back projection (FBP) has been the standard CT image reconstruction method for four decades. As shown in Figure 3, higher image noise and more artifacts are particularly noticeable in lower-dose CT imaging relying on FBP. In the 2000s, two types of iterative reconstruction algorithms were developed: model-based iterative reconstruction (MBIR) and hybrid iterative reconstruction (HIR). This type of reconstruction reduces noise and artifacts and improves both subjective and objective image quality compared to FBP at the same radiation dose [48,49]. HIR is currently the state-of-the-art reconstruction technique. Deep learning reconstruction (DLR) techniques have become increasingly popular in the past five years [50,51] (Figure 3 and Figure 4). Using artificial intelligence, DLR can generate high-quality images from lower-dose CT faster than MBIR. Super-resolution DLR (SR-DLR) has also been recently developed. SR-DLR utilizes high-resolution CT with 1024 × 1024 pixels as a model for deep learning neural networks [51] and can achieve higher-resolution images without increasing noise (Figure 5, Figure 6 and Figure 7).

Currently, image reconstruction methods vary among manufacturers of CT equipment. It is noteworthy that CT lung density measurements can be affected by scanner parameters and reconstruction methods [52]. Since 2007, CT density variability has been investigated, and efforts to reduce it have been organized by academic societies such as the Quantitative Imaging Biomarker Alliance (QIBA) [53]. As a basic premise, CT scanners should be calibrated. If CT imaging conditions are inconsistent (e.g., in multicenter studies), it is essential to standardize them first. Lee et al. introduced a convolutional neural network (CNN) architecture that can convert CT images reconstructed with one kernel into images with different reconstruction kernels, eliminating the need for a sinogram [54]. For clinical interpretation, it is recommended to use CT images with a high spatial frequency algorithm and an iterative reconstruction algorithm [55], although these images feature considerable noise, as shown in Figure 4. Generally, the histogram flattens in noisy images; for example, the percentage of low-attenuation areas (%LAA) increases [52] (Figure 6 and Figure 7).

### 3.3. Spatial Resolution

As mentioned above, high-resolution CT with 1024 × 1024 or even 2048 × 2048 matrices and slice thicknesses of 0.25 mm have already been developed [56,57]. 

**Figure 8:**
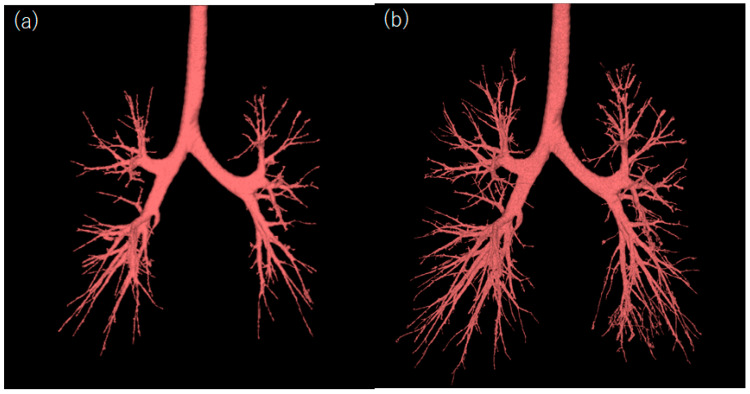
Segmented airways in different reconstruction images featuring the patient described in Figure 5. (**a**) HIR (AIDR, smooth kernel) with a matrix size of 512 × 512, and a slice thickness of 0.5 mm. (**b**) PIQE with a matrix size of 1024 × 1024 and a slice thickness of 0.5 mm. The peripheral bronchial lumen is well segmented in the PIQE image. Abbreviations: AIDR, adaptive iterative dose reduction; HIR, hybrid iterative reconstruction; and PIQE, Precise IQ Engine.

Unlike conventional CT, photon-counting CT converts individual X-ray photons directly into electric signals [46]. It can achieve high-resolution images (1024 × 1024 or 2048 × 2048 matrices and a slice thickness of 0.25 mm) with a low radiation dose [58]. Therefore, a matrix size of 1024 × 1024 and a voxel size of approximately 0.25 mm × 0.25 mm × 0.25 mm are expected to become the new standard for “thin-section CT” in the near future. Images with 1024 × 1024 matrices are excellent for visualizing peripheral bronchial lumens [4] and other peripheral lung structures [56] (Figure 1 and Figure 8). Higher-resolution images have an impact on the analysis, at least in bronchial volumetry parameters, such as total airway count (TAC) [29] and airway volume percentage (AWV%) [59].

## 4. CT Analysis in Chronic Obstructive Pulmonary Disease

### 4.1. Densitometry Analysis

We will now discuss the usefulness of quantitative assessment for each lung disease, beginning with COPD. As mentioned above, densitometric analyses of patients with COPD have been conducted since the 1980s, and various indices have been reported to be useful [10]: LAA ≤ −950 HU on full-inspiration CT (%LAA_950_) [9], LAA ≤ −910 HU on full-inspiration CT (%LAA_910_) [8], LAA ≤ −856 HU on full-expiration CT (%LAA_856_) [21], HU at the 15th percentile (Perc15) [60], and MLA [8]. These parameters show a good correlation with those of conventional pulmonary function tests, such as forced expiratory volume in one second (FEV1), FVC, and diffusing capacity of the lung for carbon monoxide (DLCO) [22]. For example, a lower Perc15 value was correlated with a lower FEV1 (r = 0.12, *p* < 0.001) [19], and %LAA showed a better correlation with %DLCO than with FEV1 [11]. 

These quantitative analyses can be more objective indicators and are suitable for evaluating disease progression. Ash et al. reported that a larger annual progression of Perc15 was associated with all-cause mortality in large cohorts [20]. Wang et al. demonstrated that ambient air pollutants were significantly associated with increased emphysema-assessed %LAA and lung function [61].

The COPD classification based on quantitative CT has also been applied in genetic research. Ito et al. found that *MMP9* expression is associated with upper lobe–predominant emphysema [62]. Moreover, Boueiz et al. identified five significant genome-wide associations with emphysema distribution [40]. 

### 4.2. Airway Assessment 

Emphysema is a key component of COPD; however, airflow limitation is caused by a combination of small airway remodeling and emphysema with varying distribution and severity [63]. Small airways (<2 mm in internal diameter) are the primary sites of airflow limitation in COPD [64,65,66]. Although thin-section CT at full inspiration cannot image the lumens of these small airway branches, Nakano et al. showed a correlation between the wall area of small airways measured through histology and that of large airways measured by CT [26]. It has been reported that the percentage wall area (WA%) correlated significantly with FVC%, FEV1% [25,67], and gas trapping, as assessed by the residual volume (RV) or its ratio to total lung capacity (TLC) [RV/TLC] [68]. 

Currently, measurement of the entire bronchial volume has also been proposed for assessing airway impairment. Smaller airway lumens reduce the number of airways visible on CT; this impaired visibility is currently assessed as the TAC [29], which is associated with FEV1, dyspnea, exercise tolerance, and future lung function decline. Tanabe et al. proposed the percentage ratio of the airway tree volume in the right upper and middle-lower lobes to the right lung volume as the AWV% for the right lung [59]. They showed that AWV% was closely correlated with FEV1 and RV/TLC in patients with COPD. In summary, central bronchial measurements are accessible and useful for evaluating pulmonary function.

### 4.3. Air Trapping on Expiratory CT

**Figure 9:**
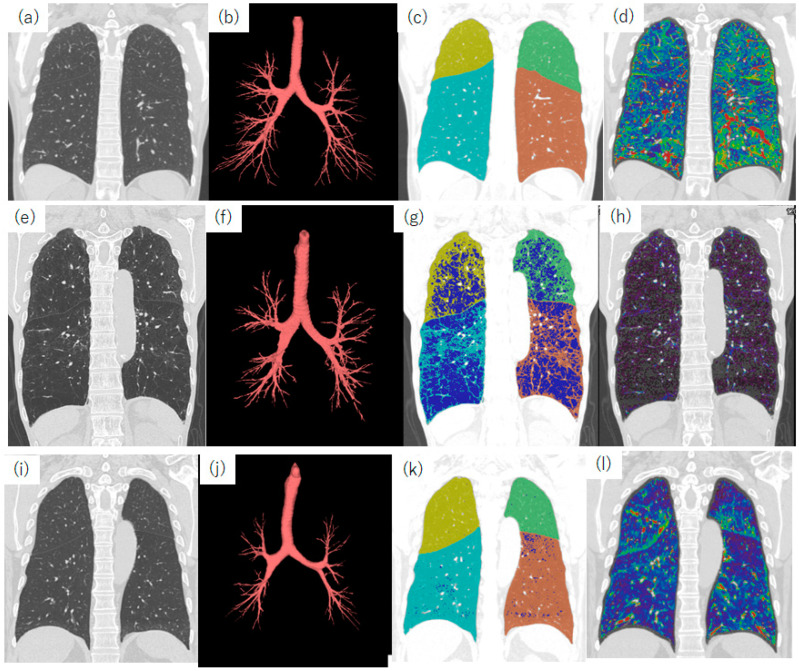
Air trapping in registered images between inspiratory and expiratory CT. (**a**–**d**) Images of a 55-year-old female never-smoker with normal respiratory function. (**e**–**h**) Images of an 81-year-old male patient with COPD who received home oxygen therapy. His lung function measurements showed an FEV1/FVC ratio of 26.6% and a %FEV1 of 42.3%. (**i**–**l**) Images of a 73-year-old male patient with COPD. His lung function measurements showed an FEV1/FVC ratio of 64.4% and a %FEV1 of 75.1%. (**a**,**e**,**i**) Coronal reconstructed standard-dose CT images. (**b**,**f**,**j**) Segmented bronchial trees. (**c**,**g**,**k**) %LAA results. Blue color indicates pixels with attenuation values below −950 HU. (**d**,**h**,**l**) Registered images between inspiratory and expiratory CT scans. The color indicates the difference in attenuation values between inspiratory and expiratory CT images. Red represents a large difference, blue indicates a small difference, and violet represents almost no difference. In the patient in the bottom row, the %LAA is 2.5%; however, marked air trapping is observed. The peripheral bronchial lumen is not segmented. These results indicate the airway subtype of COPD. Abbreviations: COPD, chronic obstructive pulmonary disease; FEV1, forced expiratory volume in one second; FVC, forced vital capacity; HU, Hounsfield unit; and LAA, low-attenuation area.

As stated earlier, it is not feasible to directly measure the lumens of small airways on full-inspiration CT scans. Nevertheless, end-expiratory CT scans can effectively demonstrate airflow limitation, including small airways, through the identification of “air trapping” [24] (Figure 9). Many studies have evaluated the presence of air trapping, which is defined as the percentage of voxels with values less than −856 or −850 HU, on expiratory CT. These values were chosen because they represented the attenuation of a normally inflated lung on inspiration, and it is assumed that a normal expiratory lung should always have a higher attenuation [69]. Schroeder et al. showed that the correlation of %LAAexp−850 on expiratory CT with FEV1 (r = −0.77) and FEV1/FVC (r = −0.84) was greater than the correlation of %LAA−950 on inspiratory CT with FEV1 (r = −0.67) and FEV1/FVC (r = −0.76) in the 4062 participants of the COPDGene study [21]. They also showed that inspiratory and expiratory volume changes decreased with increasing disease severity (*p* < 0.0001). Mets et al. found that the inspiratory-to-expiratory lung attenuation ratio had the strongest correlation with physiological air trapping [70]. 

### 4.4. Functional Small Airway Disease 

Simple threshold measurements using expiratory CT do not distinguish between gas trapping due to emphysema or to small airway disease. To solve this problem, Galbán et al. proposed a parametric response map (PRM) and the concept of functional small airway disease (fSAD) [24]. They assumed that voxels of the lung with an inspiratory CT attenuation less than −950 HU were emphysematous, whereas voxels with values greater than −950 HU on inspiration but less than −856 HU on expiration represented non-emphysematous fSAD. They analyzed follow-up CT scans in 194 patients with COPD and speculated that fSAD preceded emphysema during COPD progression. Young et al. identified two subtypes in COPD progression in the large cohort of the COPDGene study and confirmed their findings using Evaluation of COPD Longitudinally to Identify Predictive Surrogate Endpoints (ECLIPSE) data [71]. They identified two trajectories of disease progression in COPD: a “Tissue→Airway” subtype (*n* = 2354, 70.4%), in which small airway dysfunction and emphysema precede wall abnormalities of large airways, and an “Airway→Tissue” subtype (*n* = 988, 29.6%), in which wall abnormalities of large airways precede emphysema and small airway dysfunction. These subtypes were reproduced in the ECLIPSE cohort. The baseline stage in both subtypes correlated with future FEV1/FVC decline (r = −0.16 (*p* < 0.001)) in the Tissue→Airway group and r = −0.14 (*p* = 0.011P in the Airway→Tissue group) [71].

At present, FEV1/FVC on spirometry remains the most robust and widely available marker of airflow limitation, and the diagnosis of COPD requires airflow limitation, defined as a postbronchodilator FEV1/FVC < 0.7 [72]. However, CT studies have shown that considerable lung damage may already have occurred before abnormalities in FEV1/FVC became evident. Identifying individuals who eventually develop airflow obstruction consistent with a diagnosis of COPD may enable therapeutic interventions with the potential to modify the disease course [73]. The term “pre-COPD” has been proposed to identify individuals of any age with respiratory symptoms or detectable structural (e.g., emphysema) or functional (e.g., hyperinflation, reduced lung diffusing capacity, rapid FEV1 decline) abnormalities in the absence of airflow obstruction on postbronchodilator spirometry (i.e., FEV1/FVC < 0.7) [63]. Analysis of inspiratory and expiratory thin-section CTs is an essential tool for the diagnosis of pre-COPD.

### 4.5. Vessel Volume Analysis

Pulmonary vascular endothelial damage is an early step in the pathogenesis of COPD and emphysema [74]. Detection of vascular abnormalities is necessary to diagnose early stage COPD. Using non-contrast CT images, a technique to classify pulmonary arteries and veins has been developed [31]. It is well known that a loss of pulmonary capillaries is evident in pathologic sections of the emphysematous lung. Estépar et al. calculated the ratio of vascular volume to nonvascular tissue volume, which is inversely related to %LAA−950 [31]. Pistenmaa et al. segmented the pulmonary arteries and veins on non-contrast CT images and defined “pulmonary arterial pruning” as a lower ratio of small artery volume (<5 mm^2^ cross-sectional area) to the total lung artery volume [32]. They reported that greater pulmonary arterial pruning was associated with a more rapid progression of percent emphysema, even after adjusting for baseline percent emphysema and FEV1. Arterial pruning was also associated with a faster decline in the FEV1/FVC ratio. These data are consistent with those of previous studies using contrast-enhanced magnetic resonance imaging; pulmonary microvascular blood flow was reduced in patients with mild COPD, including in regions of the lungs without evident emphysema [75]. Vascular volume measurement using non-contrast thin-section CT is a minimally invasive and useful tool for understanding the pathophysiology of COPD.

## 5. CT Analysis of ILD 

### 5.1. Automatic Extraction of Various ILD Lesions in Thin-Section CT

Quantitative CT evaluation is essential for the diagnosis and management of patients with ILD. Various CT patterns are observed in these patients, including ground-glass opacities, consolidation, reticulation, honeycomb pattern, and emphysema. Therefore, dedicated software is required to classify these lesions. Table 2 summarizes the available systems.

Computer-Aided Lung Informatics for Pathology Evaluation and Rating (CALIPER), developed at the Biomedical Imaging Resource (Mayo Clinic, Rochester, MN, USA), is one of the most well-known software packages [81], but the principles on which it is based have not been disclosed in detail. We previously developed a classification system, the Gaussian Histogram Normalized Correlation (GHNC), which divides pixels on CT images into several patterns based on local histograms in original and differential images [78]. This GHNC approach not only can extract honeycomb patterns, but it can also detect subpleural fibrosis in patients with IPF. In a study comparing histological specimens with GHNC results, GHNC detected subpleural fibrosis, which is a characteristic finding of usual interstitial pneumonia (UIP)-pattern fibrosis [78]. Subpleural fibrosis is significantly correlated with prognosis in patients with lung cancer [87].

### 5.2. CT Lung Volume in Interstitial Lung Disease

Lung volume is a basic biomarker of ILD. A strong correlation exists between CT-derived automated lung volume, TLC, and FVC [88,89]. Si-Mohamed et al. reported that CT lung volume was correlated with FVC (r: 0.86) and TLC (r: 0.84) (*p* < 0.0001). They also reported that the median annual lung volume loss over 5.03 years was 155.7 mL in IPF versus 50.7 mL in non-IPF (*p* < 0.0001) [89].

Lung volume can be measured with relative ease using 3D thin-section CT in patients with COPD because the lungs can be considered as air-containing material exhibiting low attenuation. However, in ILD, a simple density mask approach cannot effectively distinguish the lung from the chest wall due to lesions with high attenuation, such as consolidation and fibrosis [90]. Therefore, it is recommended to employ dedicated software for the automatic segmentation of the lung, as mentioned above [78,82,85,91].

The lung volume measured on inspiratory CT showed a good correlation with TLC measured using plethysmography despite being smaller [89,92,93], especially in patients with COPD, due to obstructive impairment. Another reason for this difference is the body position during testing. Conventional pulmonary function tests are typically performed with the patient in the seated position, whereas thin-section CT is usually performed in the supine position. CT lung volumes in a healthy cohort have been reported in the United States using data from non-Hispanic white individuals from the COPDGene cohort [94], as well as in Korean patients [95].

### 5.3. Evaluation of Disease Progression in Patients with ILD

Thin-section CT and quantitative analysis are essential tools for evaluating disease progression in patients with ILD. The effectiveness of antifibrotic agents has been demonstrated in various fibrotic ILDs [96,97], and the identification of disease progression is an important factor in determining treatment, especially in progressive fibrosing ILDs. In recent guidelines, progressive pulmonary fibrosis was defined as at least two of the three criteria (worsening symptoms, radiological progression, and physiological progression) occurring within the past year, with no alternative explanation in a patient with an ILD other than IPF [98].

**Figure 10:**
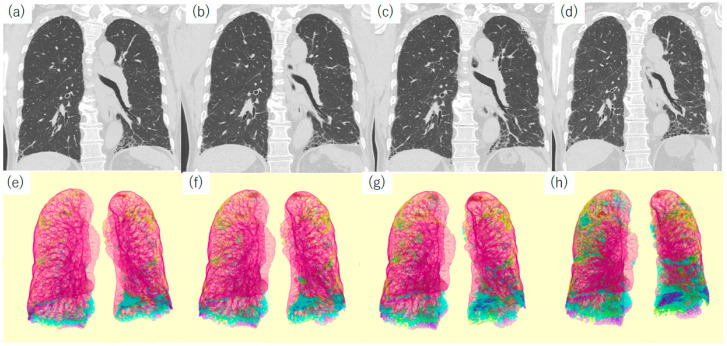
CT images and their segmented images of a 70-year-old male patient with IPF (UIP pattern was proven by surgical lung biopsy.) (**a**,**e**) Initial CT, (**b**,**f**) CT taken two years later, (**c**,**g**) CT obtained four years later, and (**d**,**h**) CT after six years. All CTs were acquired at a standard dose. Segmentation was performed using QZIP-ILD (Ziosoft Inc.). Segmentation color codes: pink, normal lung tissue; green, GGO, blue, reticulation; yellow, consolidation with traction bronchiectasis; and violet, honeycomb pattern. In the initial CT image, a small amount of honeycomb pattern can be observed bilaterally at the lung base. Abbreviations: CT, computed tomography; GGO, ground-glass opacity; IPF, idiopathic pulmonary fibrosis; and UIP, usual interstitial pneumonia.

**Figure 11:**
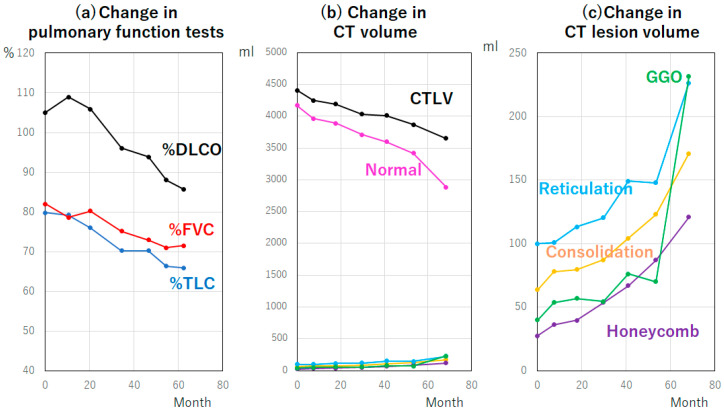
Changes in pulmonary function tests and CT lesion volume. The panels show the changes in pulmonary function tests (**a**), CT volume (**b**) and CT lesion volume (**c**) in the patient described in Figure 10. Abbreviations: CTLV, CT lung volume; DLCO, diffusing capacity of the lung for carbon monoxide; FVC, forced vital capacity; GGO, ground-glass opacity; and TLC, total lung capacity.

CT is one of the important tests for evaluating disease progression; however, Oldham et al. reported that the description of “disease progression” or “worsening” on daily radiologists’ reports delayed declining in FVC or DLCO [99]. One of the reasons for this observation is the shrinkage of ILD lesions. UIP-pattern fibrosis is accompanied by alveolar collapse, and the volume of the lesion decreases [100] (Figure 10 and Figure 11). Lung shrinkage is also observed in systemic sclerosis, which shows nonspecific interstitial pneumonia (NSIP)-pattern fibrosis in typical cases. Chassagnon et al. demonstrated this lesion shrinkage visually and quantitatively using Jacobian maps [101]. 

**Figure 12:**
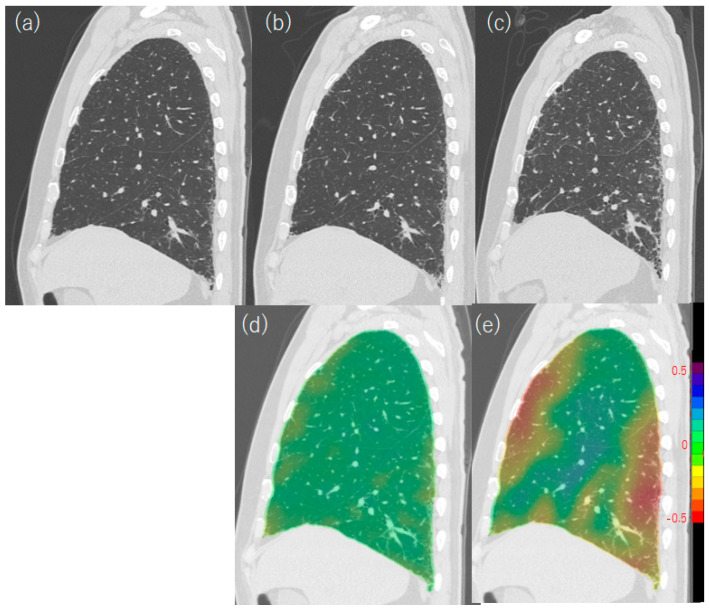
Jacobian maps of CT images at different time points in a 70-year-old male patient with IPF (UIP pattern was proven by surgical lung biopsy). (**a**) Initial CT, (**b**) CT after one year, (**c**) CT after six years, (**d**) Jacobian map after one year, and (**e**) Jacobian map after six years. Follow-up CT images were elastically registered to align with baseline images, enabling the calculation of deformation maps using QZIP-ILD (Ziosoft Inc. (Tokyo, Japan)). Each voxel was matched to the corresponding baseline lung scan in the follow-up examination, and the change in voxel size was represented using a color map, with red indicating shrinkage. In the Jacobian map, faint shrinkage (yellow) is observed in the subpleural region after one year (**d**), whereas the ventral and dorsal subpleural regions show marked shrinkage (red) after six years (**e**). The lung height decreased in the follow-up images. CT, computed tomography; IPF, idiopathic pulmonary fibrosis; and UIP, usual interstitial pneumonia.

Due to lesion shrinkage, the increase in the volume of lesions is lower than the decrease in the volume of the entire lung and the normal lung [79], as shown in Figure 11. Therefore, it is important to note decreases in CT and normal lung volumes when evaluating disease progression [89]. The Jacobian map and elastic registration between baseline and follow-up CT images enabled the quantification of voxel stretching and shrinkage, represented as a color map. This approach aided in the identification of local shrinkage and disease progression in patients with ILD (Figure 12) [101]. In daily clinical practice, measuring lung height using sagittal images can serve as a valuable surrogate marker that correlates with CT lung volume [88] (Figure 12).

### 5.4. Quantification of High-Attenuation Areas on Thin-Section CT

In addition to dedicated software, conventional densitometry can be applied to ILDs [102]. If a specific range of CT values is considered normal for the lungs, areas with high CT values are regarded as lesions, as is the case of fibrosis, and areas with low CT values are considered indicative of emphysema. Then, the volume ratios of normal and diseased lungs for the total CT lung volume can be determined. Densitometric analysis is a popular approach for ILD. We searched the PubMed database for all original research articles published in English between January 1990 and June 2023, including those on ILD, CT, and quantitative analysis. In total, 207 original articles were identified, of which 136 utilized densitometric analyses. For example, Best et al. found a correlation between kurtosis and FVC (r = 0.53) in patients with IPF [18]. They also showed that MLA (*p* = 0.003), skewness (*p* = 0.001), and kurtosis (*p* = 0.001) deteriorated with fibrosis progression, as determined by radiologists in patients with IPF [103]. 

Quantification of HAAs has been applied in large cohorts to screen for ILD. In large cohort studies (such as screening for lung cancer), awareness has increased regarding the clinical importance of incidentally detected interstitial lung abnormalities (ILAs) on non-contrast chest CT scans [104]. An ILA refers to a subtle or mild parenchymal abnormality identified in more than 5% of lungs on CT scans in patients where ILD was not clinically suspected previously [105]. ILAs tend to progress slowly over time and are independent risk factors for death [106]. In previous studies, radiologists determined the presence of ILAs [107]; however, densitometric analysis offers a more objective approach to their detection. Easthausen et al. measured HAAs in 3110 participants in the MESA study and developed a prediction model for HAAs in healthy never smokers [108]. They also observed increased HAA levels in participants with ILA and exertional dyspnea. Kim et al. measured %HAA in the MESA study cohort, and each *MUC5B* risk allele (T) was associated with an increase in HAAs of 2.60% (95% confidence interval: 0.36–4.86) in the course of 10 years [109].

In addition, we would like to discuss the syndrome of combined pulmonary fibrosis and emphysema (CPFE) [110]. CPFE is characterized by the coexistence of pulmonary fibrosis and emphysema, sharing pathogenic pathways and presenting unique considerations related to disease progression, along with an increased risk of complications such as pulmonary hypertension and lung cancer, and increased mortality. CT plays a vital role as a diagnostic tool that enables the quantification of emphysema and fibrosis. However, distinguishing honeycombs from emphysematous fibrosis remains challenging [111]. We believe that the conventional densitometric approach is suitable for CPFE, and several researchers have utilized densitometric analysis for CPFE [112,113]. We advocate that conventional densitometric analysis should be considered when assessing CPFE.

### 5.5. Identification of UIP Patterns

Some previous studies have focused on the identification of honeycombs [114] because they are essential for the CT-based diagnosis of UIP. These studies showed worse prognosis in patients with honeycombs in various ILDs, such as in patients with lung cancer [115] or rheumatoid arthritis [116]. However, recent evidence suggests that patients with a probable UIP pattern (without honeycombs) exhibit similar disease behaviors and clinical courses to those in patients with honeycombs [117]. Considering treatment with antifibrotic drugs, the detection of progressive fibrosis, with or without honeycombs, has become more important in ILDs other than IPF, including collagen vascular disease and hypersensitivity pneumonitis [118,119]. 

In UIP, fibrosis occurs in the perilobular area of the secondary pulmonary lobule [120]. Although the area along the large airways and vessels is perilobular, many other lesions occur along the bronchovascular bundles, including other types of fibrosis such as NSIP, bronchitis, and vasculitis. Distinguishing these lesions from UIP-pattern fibrosis on thin-section CT images is challenging. 

**Figure 13:**
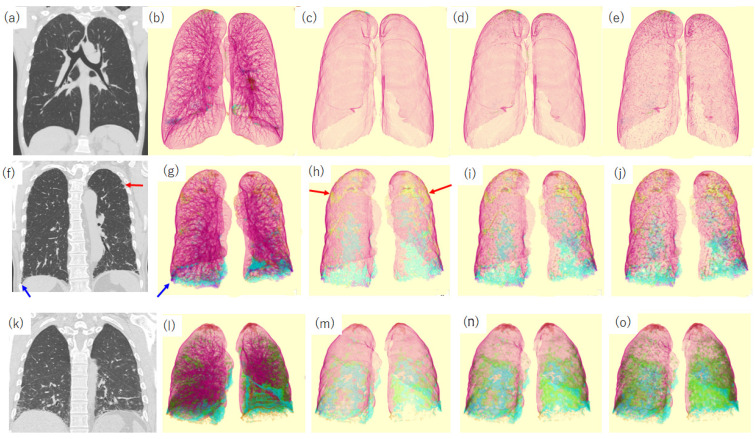
Coronal CT image and quantification CT images of the lung surface. (**a**–**e**) Images of a 41-year-old male never-smoker with normal respiratory function. (**f**–**j**) Images of a 70-year-old male patient with IPF (images from the same patient are shown in Figure 10, Figure 11 and Figure 12). (**k**–**o**) Images of a 41-year-old man with pathologically proven idiopathic NSIP. (**a**,**f**,**k**) Coronal CT images obtained at standard dose. (**b**,**g**,**l**) Segmented images of the entire lung. (**c**,**h**,**m**) outer part with a 2 mm width. (**d**,**i**,**n**) outer part with a 5 mm width. (**e**,**j**,**o**) outer part with a 10 mm width. All segmentations were performed using QZIP-ILD. Segmentation color codes are shown in Figure 10. In healthy individuals, no structures are observed in the outer part, with a width of 2 mm. (**c**) Peripheral pulmonary vessels and bronchi are recognized in the outer part with a 10 mm width. (**e**) In patients with UIP, a violet pattern, indicating a honeycomb is observed in the bilateral lung base (blue arrows in **f** and **g**). Additionally, a yellow pattern corresponding to subpleural fibrosis is observed on the surface of the bilateral upper lobes (red arrows in **f** and **h**). In patients with NSIP, the upper lobe surface area shows a normal pattern. Abbreviations: CT, computed tomography; IPF, idiopathic pulmonary fibrosis; NSIP, nonspecific interstitial pneumonia; and UIP, usual interstitial pneumonia.

Figure 13 shows the segmented images of the outer part of the lung. As shown in Figure 1, anatomically, the centrilobular region is situated approximately 3–5 mm away from the adjacent lobular border [121]. In the subpleural region, bronchovascular bundles are absent in the normal lung on CT (Figure 1 and Figure 13c). Consequently, interstitial lesions observed within 2–3 mm of the pleura in the subpleural region are highly likely to exhibit a UIP pattern (Figure 13h). Hunninghake et al. reported that CT findings of lower lung honeycombs and upper lung subpleural irregular lines were most closely associated with a pathological diagnosis of UIP [122]. In NSIP, subpleural sparing was a characteristic finding [123] (Figure 13m). Our previous study showed that small subpleural opacities on CT corresponded to histological UIP and that the fibrotic lesion volume ratio in the subpleural area was associated with a worse prognosis [87]. Umakoshi et al. also reported that increased %HAA in subpleural regions was significantly correlated with decreased DLCO [124]. We believe that the evaluation of the lung surface will be useful for detecting UIP-pattern fibrosis.

## 6. Thin-Section CT Analysis for COVID-19 Pneumonia

### 6.1. CT Findings of COVID-19 Pneumonia

**Figure 14:**
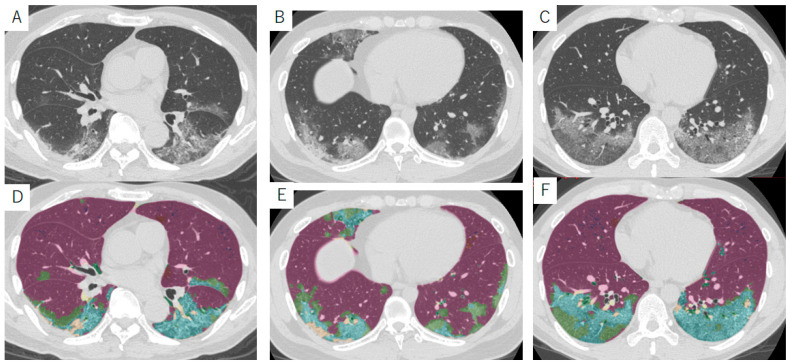
Typical CT images and their segmented images of patients with COVID-19 pneumonia. All segmentations were performed using QZIP-ILD. (**A**,**D**) Images from a 70-year-old man. The lesion area is 21% of the lung area in this slice. (**B**,**E**) Images from a 28-year-old man. The lesion area in this slice is 25% of the lung area in this slice. (**C**,**F**) Images from a 39-year-old man. The lesion area in this slice is 30% of the lung area in this slice. All patients were admitted and required oxygen therapy. These figures were used in Kanagawa Prefecture, Japan, during the COVID-19 pandemic as a reference to determine the need for hospital admission. Abbreviations: COVID-19, coronavirus disease 2019.

During the coronavirus disease 2019 (COVID-19) pandemic, a large number of CTs have been obtained for the detection of lung abnormalities and their severity assessments. The typical CT pattern of COVID-19 pneumonia includes bilateral peripheral GGO abnormalities, crazy-paving pattern, and consolidation [125] (Figure 12). The initial lung findings on chest CT are small subpleural GGOs that grow larger with a crazy-paving pattern and consolidation [126] (Figure 13). Lung involvement increases to consolidation up to two weeks after symptom onset [127,128]. Many CT studies have shown that a greater lesion extent on CT correlates with disease severity during COVID-19 pneumonia [129]. In initial studies, the extent was evaluated visually [130], but many dedicated software packages have been developed, including some using artificial intelligence [131]. Densitometric analysis has also been used to evaluate the extent of pulmonary lesions [132,133,134]. Quantitative CT results have been widely used in clinical settings to determine the appropriate level of care and management strategies, including the need for hospitalization or intensive care. Figure 12 shows CT images and quantitative results of patients with COVID-19, which were used in Kanagawa Prefecture in Japan in 2021 as a reference to determine the need for hospitalization (Figure 14).

### 6.2. Estimation of Respiratory Function Using CT in COVID-19 Pneumonia

**Figure 15:**
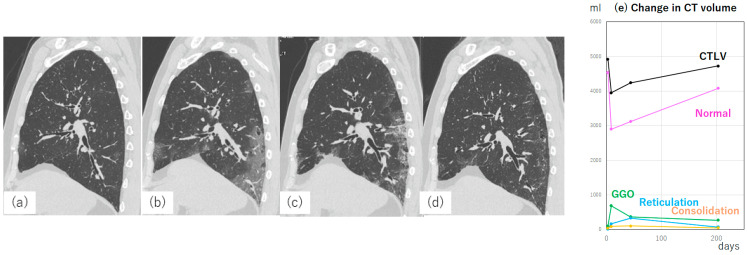
Sagittal CT images from a 74-year-old male patient with COVID-19 pneumonia and their quantitative analysis. CT images were obtained at (**a**) 3 days, (**b**) 9 days, (**c**) 44 days, and (**d**) 6 months from symptom onset. (**e**) The change in CT lung volume, normal and each lesion measured by QZIP-ILD. The patient was admitted to our hospital with a cough and fever. (**a**) The PCR test for COVID-19 was positive. Hypoxemia progressed rapidly, and the patient required oxygen administration at 10 L/min O_2_. (**b**) The CT lung volume decreased by approximately 1000 mL, and the lung height on the sagittal image decreased. Abbreviations: COVID-19, coronavirus disease 2019; CTLV, CT lung volume; GGO, ground-glass opacity; and PCR, polymerase chain reaction.

In the context of COVID-19, the use of pulmonary function tests and other clinical evaluations is limited. Thin-section CT provides valuable insights into respiratory function [135]. For example, a greater extent of disease indicates decreased aeration of the lungs. We reported smaller secondary lobules in COVID-19 pneumonia lesions observed on high-resolution CT with a 1024 × 1024 matrix size and 0.25 mm slice thickness, suggesting alveolar collapse in these lesions [5]. This hypothesis is supported by experimental results showing the downregulation of surfactant expression in alveolar type 2 cells infected with severe acute respiratory syndrome coronavirus 2 (SARS-CoV-2) [136]. Decreased lung volume is often observed in patients with severe COVID-19 [137] (Figure 15). 

The pulmonary blood volume (BV) in vessels less than 5 mm^2^ (i.e., BV5, corresponding to a diameter of 1.25 mm) is reduced on thin-section CT in patients with COVID-19, whereas the vascular volume is increased in vessels within the 5–10 mm^2^ range (i.e., BV5–10) and larger than 10 mm^2^ (BV10) [138]. The BV5% value, calculated as the proportion of BV5 to total BV, was found to be a prognostic factor for adverse outcomes (intubation or mortality) in patients with COVID-19 [139]. In thin-section CT scans of COVID-19 pneumonia, bilateral distribution and subsegmental vessel enlargement are usually observed in clinical situations [85,140,141]. These vascular abnormalities are consistent with the results of dual-energy CT [142,143] and microvascular observations using video microscopy [143]. These vascular changes can reflect the failure of physiological hypoxic vasoconstriction caused by dysfunctional and diffuse inflammation or secondary vascular dilatation (i.e., congestion) proximal to SARS-CoV-2-affected microvessels due to microvascular thrombosis [144]. These vascular and parenchymal abnormalities result in V/Q mismatches, shunts, and marked hypoxemia in patients with severe disease. CT is an essential tool for identifying COVID-19 pneumonia patients at risk of deterioration.

## 7. Conclusions

Thin-section CT is a widely adopted imaging modality that has enabled various quantitative analyses using the volume and attenuation values of the lungs, airways, and vessels. Many of these analyses can be readily performed using commercially available equipment. Previous studies have demonstrated that the results of these analyses are correlated with conventional respiratory function and patient prognosis in a variety of diseases. Therefore, we strongly advocate for the incorporation of these analyses into routine clinical practice. Even if challenges in their implementation still subsist, these results should be carefully considered and applied to benefit patients in clinical settings. 

Thin-section CT is an accessible and well-established tool for evaluating respiratory function.

## Figures and Tables

**Table 1:** Indices of thin-section CT for measuring pulmonary function.

Index	Method	Target Lesion	Reference
Lung volume	Lung volume measured in thin-section CT	COPD and ILD	[7]
%LAA	Volume ratio of LAAs below a certain threshold (usually −950 HU) in inspiratory CT	Emphysema	[8,9,10,11,12]
%HAA	Volume ratio of HAAs over a certain threshold (usually −700 HU) in inspiratory CT	ILD	[13,14]
LAC	Low-attenuation clusters analysis	Emphysema	[15]
Fractal dimension	Size distribution of clusters of emphysematous regions	Emphysema	[16,17]
MLA	Mean lung attenuation in inspiratory CT	COPD and ILD	[18]
Perc15	HU at the 15th percentile of the histogram in inspiratory CT	Emphysema	[19,20]
Skewness and kurtosis	Histogram indices in inspiratory CT	ILD	[18]
Air trapping	LAAs below a certain threshold (usually −856 HU) in full-expiration CT	Small airway disease	[21,22]
PRM and DPM	Inspiratory and expiratory CT images are registered, and each voxel is classified as emphysema, gas trapping, or normal	Identification of fSAD and pre-COPD	[23,24]
WA%	Wall area percentage defined as Ao − Ai/Ao × 100 (Ai; luminal area, Ao; total area of the airways)	Airway narrowing	[25,26]
Pi10	Pi10 calculation: The square root of the wall area is plotted against the internal perimeter for each measured airway.	Airway narrowing	[26,27,28]
TAC	Total airway count; the sum of all airway segments from the segmented airway tree	Central airway narrowing	[28,29]
%CSA	Total area of small pulmonary vessels (usually less than 5 mm in diameter) in 2D images	Pulmonary vessels	[30]
Vessel volume	Vessel volume in 3D images	Pulmonary vessels	[31,32]
Vessel-related structures	Vessel volume measured using CALIPER	Pulmonary vessels in ILD	[33,34]

Abbreviations: COPD, chronic obstructive pulmonary disease; CALIPER, Computer-Aided Lung Informatics for Pathology Evaluation and Rating; CSA, cross-sectional area; CT, computed tomography; DPM, disease probability measure; fSAD, functional small airway disease; HAA, high-attenuation area; HU, Hounsfield unit; ILD, interstitial lung disease; LAA, low-attenuation area; LAC, low-attenuation cluster; MLA, mean lung attenuation; PRM, parametric response map; TAC, total airway count; and WA, wall area.

**Table 2:** Segmentation systems for interstitial lung diseases.

System	Method	References
Adaptive multiple feature method (AMFM)	Texture-based method with 17 texture parameters	[76,77]
Gaussian Histogram Normalized Correlation (GHNC)	Use of local histograms in original and differential images	[78,79,80]
Computer-Aided Lung Informatics for Pathology Evaluation and Rating (CALIPER)	Biomedical Imaging Resource (Mayo Clinic, Rochester, MN, USA)	[33,34,81]
Novel artificial intelligence-based quantitative CT image analysis software (AIQCT)	Deep Learning-based Texture Analysis (Fujifilm corporation, Tokyo, Japan)	[82]
CT Lung Parenchyma Analysis	Three-dimensional machine learning for CT texture analysis (Canon Medical Systems, Tochigi, Japan)	[83,84]
QZIP-ILD	Deep Learning-based Texture Analysis (Ziosoft, Inc., Tokyo, Japan).	[85]
AVIEW Lung Texture interstitial lung abnormalities	Deep Learning-based Texture Analysis (Coreline, Seoul, Republic of Korea).	[86]
Data-driven texture analysis (DTA)	Convolutional neural network algorithms	[42]

## Data Availability

These data are unavailable due to privacy or ethical restrictions.
